# TLR4/NF-κB Signaling Induces GSDMD-Related Pyroptosis in Tubular Cells in Diabetic Kidney Disease

**DOI:** 10.3389/fendo.2019.00603

**Published:** 2019-09-19

**Authors:** Youliang Wang, Xuejing Zhu, Shuguang Yuan, Si Wen, Xuemei Liu, Chang Wang, Zhong Qu, Jun Li, Hong Liu, Lin Sun, Fuyou Liu

**Affiliations:** ^1^Department of Nephrology, Hunan Key Laboratory of Kidney Disease and Blood Purification, The Second Xiangya Hospital of Central South University, Changsha, China; ^2^Changsha Central Hospital, Changsha, China

**Keywords:** diabetic kidney disease, tubular injury, Toll-like receptor-4, pyroptosis, gasdermin-D

## Abstract

Gasdermin D (GSDMD) has been proven to be a key protein in the activation of pyroptosis. Pyroptosis of renal tubular epithelial cells contributes to the progression of tubular injury in kidney diseases. However, it remains elusive whether and how GSDMD is involved in the regulation of diabetic kidney disease (DKD). In this study, we found that tubular injury is accompanied by the up-regulation of Toll-like receptor 4 (TLR4) and GSDMD in patients with diabetic kidney disease. In addition, we discovered that the expressions of cleaved Caspase-1, active N-terminal fragments of GSDMD (GSDMD-NT), IL-18, and the secretion of IL-1β also increased in the kidneys of db/db mice. These changes were partially ameliorated following intraperitoneal injection of TAK-242, an inhibitor of TLR4. Similar results were observed in human tubular cells (HK-2) subjected to high-glucose (HG) conditions and treated with TAK-242 or parthenolide (inhibitor of NF-κB) by Western blot, Enzyme-linked immunosorbent assay (ELISA), and flow cytometry. These results indicated that TLR4/NF-κB signaling could induce GSDMD-mediated pyroptosis in tubular cells in DKD.

## Introduction

Recently, tubular injury induced by high glucose has been taken into consideration to be an independent risk factor for the progression of diabetic kidney disease (DKD) (1, 2). Pyroptosis is a programmed cell death mediated by the activation of caspase family, such as caspase-1, in the pathogenesis of disorders characterized by excessive cell death and inflammation. This form of cell death is accompanied by water influx, cellular swelling, osmotic lysis, activation of NLRP3 inflammasome, and the release of pro-inflammatory cytokines, such as IL-1β and IL-18 (3, 4). Researchers have found that pyroptosis could be induced by hyperglycemia in diabetic cardiomyopathy (5). In addition, several reports have proved that pyroptosis of renal tubular epithelial cells is an indispensable process for contrast-induced acute kidney injury and renal ischemia-reperfusion injury (6, 7), indicating the contribution of pyroptosis in the progression of tubular injury in kidney diseases. However, few studies have focused on the role of pyroptosis in tubular cells of diabetic kidney disease.

Toll-like receptor 4 (TLR4) is one of TLRs family which plays a fundamental role in the activation of innate and adaptive immune responses (8, 9). It has been reported to be involved in the pathogenesis of acute and chronic renal disorders such as acute kidney injury (AKI), renal fibrosis, and DKD (10, 11). TLR4 typically signals through its downstream partner MyD88 to activate the nuclear factor-κB (NF-κB) pathway, leading to ROS and cytokine production. In HG-stimulated podocytes, TLR4 was found to activate NF-κB, increasing the release of pro-inflammatory cytokines and chemokines (12, 13). All of these findings indicate that TLR4/NF-κB signaling plays a crucial part in the progression of DKD. TLR4 was also proved to induce Caspase-11 expression and Caspase-1 activation, resulting in cellular pyroptosis and increased release of pro-inflammatory cytokines (14). Recently, gasdermin D (GSDMD), which belongs to a family of gasdermins, mainly expressed in the skin and mucosa, has been proven to be a key protein in the activation of pyroptosis. It could be cleaved by activated inflammatory caspases such as Caspase-1. The active N-terminal fragment oligomerizes to form membrane pores which cause leakage of the pro-inflammatory cytokines. But the role of GSDMD in the TLR4-induced pyroptosis in DKD patients is not clear.

Our aim in this study is to explore the effect of the TLR4/NF-κB pathway on the pyroptosis of renal tubular epithelial cells in DKD, and to investigate the role of GSDMD in this TLR4/NF-κB-mediated pathway.

## Materials and Methods

### Main Reagents and Materials

Human kidney proximal tubular epithelial cells (HK-2) were a cell line purchased from the American Type Culture Collection (ATCC, USA). Antibodies were from the following sources: polyclonal anti-TLR4 (19811-1-AP), β-actin (20536-1-AP), β-tubulin (10094-1-AP), GSDMD for HK2 (20770-1-AP), and Caspase 1 (22915-1-AP) were from Proteintech (Wuhan, China); Caspase-1 p20 (ab1872), human GSDMD-N (ab210070), and mouse GSDMD-N (ab219800) were from Abcam; NF-κB p65 (#3034; CST) and Phospho-NF-κB p65 (ser536) (#3031; CST) antibody were from Cell Signaling Technology. All secondary antibodies for western blot were from Proteintech (Wuhan, China). TAK-242 was from MedChem Express (HY-11109; USA), and parthenolide was from Sigma (USA). FAM-FLICA Caspase 1 Assay Kit was from ICT (USA). IL-1β ELISA kit was from Huamei (Wuhan, China). Other reagents, including DMEM/F12 medium, bovine serum albumin (FBS) and trypsin were obtained from Gibco (USA).

### Morphological Analysis of Kidney

Thirty patients with DKD and 13 non-DKD controls were recruited for this study. We chose patients without diabetes (non-DKD) who clinically manifest as mild hematuria or proteinuria patients, have kidney biopsies diagnosed as minor glomerular lesions, and do not incorporate with other glomerular diseases such as IgA nephropathy or membranous nephropathy as controls. Kidney biopsy tissues were stained with periodic acid-silver methenamine (PASM). A semiquantitative scoring system was used to evaluate the tubulointerstitial lesion index and tubular damage according to guidelines in 2010 by Tervaet et al. (1, 15). The percentage area of tubular injury was estimated by a pathologist in a blinded manner according to the following criteria: tubular dilation, detachment of brush border, tubular atrophy, and interstitial fibrosis in every high-power field across whole tissue sample (magnification, × 200). All patients did not use adrenal cortical hormones or immunosuppression. All of the DKD patients use insulin combined with metformin to control blood glucose. The institutional review board and the administrators of the Department of Nephrology in Second Xiangya Hospital approved the protocol for this study. Written informed consent was obtained from all the participants.

### Animal Experimental Design

A total of 5 adult male db/m mice and 10 adult male db/db mice at 16 weeks of age were divided into three groups of 5 animals each. Male db/m mice served as a control (db/m group). Db/db mice received an intraperitoneal injection with the vehicle alone (db/db group), or TLR4 inhibitor for 7 consecutive days (3 mg/kg) (dbdb + TAK242 group). The mice were sacrificed at 17 weeks following administration. The kidney tissue of the mice was processed as described previously (16). The Institutional Animal Experimentation Ethics Committee as described above approved the experimental animal protocols.

### Cell Culture

Human kidney proximal tubular epithelial cells (HK-2) were cultured in DMEM/F12 medium, supplemented with 10% FBS, penicillin 1 × 10^5^ U/L, and streptomycin 100 mg/L and incubated at 37°C in a 5% CO_2_ environment. Until an 80–90% confluence was reached, the cells were exposed to different concentrations of D-glucose and TLR4/NF-κB blockers, and divided into the following groups: (A) 5.5 mM D-glucose (control/5.5Glu group), (B) 30 mM D-glucose (30Glu group), (C) 30 mM D-glucose and TLR4 inhibitor (TAK-242) (30Glu+TAK-242 group), and (D) 30 mM D-glucose and NF-kB blocker (parthenolide) (30Glu + parthenolide group).

### Immunohistochemistry (IHC)

Renal tissue sections for immunostaining were deparaffinized, rehydrated, and antigen retrieval in a microwave oven. Immunohistochemistry was performed using anti-TLR4 (1:100), GSDMD (1:100, Proteintech), GSDMD-NT (1:800, abcam) and IL-18 (1:100, abcam) antibody as the primary antibodies, followed by a secondary antibody. Then slides were visualized by using a DAB detection kit according to the manufacturer's instructions, and the tissue specimens were examined by light microscopy. The TUNEL staining was used to detect cell death conditions following the manufacturer's instructions.

### Western Blotting (WB)

After inventions, the whole cell lysate of HK-2 was obtained using RIPA buffer (Beyotime, Shanghai, China) and cocktail (Roche Diagnostics, Mannheim, Germany). Cell lysates were then sonicated to release nuclear proteins, spun at 12,000 rpm at 4°C, and stored at −80°C. The protein concentration was quantified by the BCA protein assay (Beyotime, Shanghai, China). Then the samples were separated in 10% SDS-polyacrylamide gels, transferred onto polyvinylidene difluoride membranes (Millipore, MA, USA), and incubated overnight at 4°C with primary antibodies. After overnight incubation, membranes were washed 3 times, after which they were incubated with secondary antibodies (anti-mouse/rabbit IgG, Proteintech, Wuhan, China) for 1 h at room temperature. The blots were subsequently detected using ECL (Millipore, MA, USA). Primary antibodies used in this experiment were β-actin (1:3,000, Proteintech), β-tubulin (1:3,000, Proteintech), polyclonal anti-TLR4 (1:1,000, proteintech), human GSDMD-NT (1:1,000, Abcam), mouse GSDMD-NT (1:1,000, Abcam), caspase-1 (1:1,000, Proteintech), caspase-1 p20 (1:1,000, Abcam), NF-κB p65 antibody (1:1,000, CST), and phospho-NF-κB p65 (ser536) antibody (1:1,000, CST).

### Flow Cytometry Analysis of Pyroptosis

Cell pyroptosis was measured with FAM-FLICA Caspase-1 Assay Kits (ICT, USA). According to the manufacturer's instructions, cells were incubated with 40 μl phosphate buffered saline (PH = 7.4) containing 10 μl FLICA at 37°C protected from light for 30 min and 5 μL propidium iodide. Cell pyroptosis was analyzed on a FACScalibur flow cytometer (BD Biosciences, San Jose, USA). The double-positive cells were considered to be pyroptotic cells.

### Data Analysis

Statistical analysis was carried out using the SPSS 20 software. Quantitative Data are expressed as means ± SEM (standard error of the mean). Statistical differences among groups were analyzed by One-way ANOVA, and two-tailed *P*-values were reported. Spearman's correlation was used to evaluate the correlations among each histopathological and clinical finding. *P-*values < 0.05 were considered statistically significant.

## Results

### Clinical Characteristics and Kidney Morphological Changes in Patients With DKD

**Figure 1:**
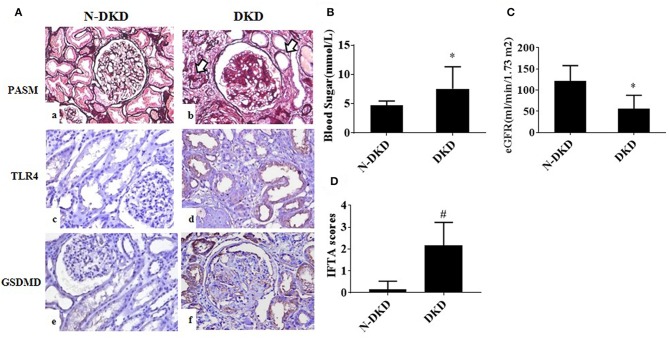
TLR4 and GSDMD were increased in renal tubules of DKD patients. **(A)** PASM staining in renal biopsy specimens of patients with Non-DKD(N-DKD) (a) and DKD (b) (arrow indicates focal tubular atrophy and interstitial fibrosis) (magnification ×200). Immunohistochemistry staining demonstrated the expression of TLR4 in renal biopsy tissues of patients with DKD (d) vs. N-DKD (c), and expressions of GSDMD in renal biopsy tissues of patients with DKD (f) versus N-DKD (e). **(B–D)** Blood glucose, eGFR levels, and IFTA scores in DKD and NDKD patients. Values are means ± SEM. *P < 0.05, ^#^*P* < 0.01.

Patients with DKD exhibited higher blood glucose and 24 h urine protein levels compared with non-DKD patients, as shown in Table 1. PASM staining showed focal tubular atrophy and interstitial fibrosis in DKD patients (arrow) compared with those in non-DKD patients (Figure 1Aa,b). TLR4 and GSDMD expression was examined in paraffin-embedded sections of human diabetic kidney tissues by immunohistochemical staining. Both enhanced TLR4 (Figure 1Ac,d) and GSDMD (Figure 1Ae,f) expressions were observed predominantly in proximal tubules of DKD patients.

**Table 1:** Clinical characteristics of the patients with DKD and non-DKD.

**Clinical data**	**Control**	**DN**
Age (years)	25.08 ± 3.223	46.03 ± 2.112^#^
Sex (male/female)	4/9	16/14
Duration (month)	26.88 ± 7.501	63.33 ± 11.85
Hematuria (%)	6 (46.2%)	21 (70%)
Hb (g/L)	130.9 ± 3.736	109.5 ± 4.247^#^
Albumin (g/L)	31.82 ± 3.316	24.41 ± 1.391^*^
Total cholesterol (mmol/L)	5.967 ± 0.871	5.82 ± 0.3329
Triglyceride (mmol/L)	1.302 ± 0.3514	2.276 ± 0.2289^*^
Scr (μJmol/L)	65.45 ± 8.992	144.3 ± 17.66^#^
BUN (mmol/L)	5.428 ± 0.6621	8.466 ± 0.6959^*^
UA (μJmol/L)	296 ± 29.34	362 ± 16.17^*^
Urine protein (mg/24 h)	832 ± 503.3	5978 ± 970.1^#^

*Hb, Hemoglobin; Scr, serum creatinine; BUN, blood urea nitrogen; UA, uric acid*.

* p < 0.05, vs. control;

# *p < 0.01, vs. control. Values are the mean ± SE*.

To further determine the relationship between GSDMD expression and diabetic nephropathy and tubular injury, correlation analyses were made. Results showed that GSDMD expression was positively correlated with TLR4 expression (*r* = 0.988, *P* = 0.000), proteinuria (*r* = 0.423, *P* = 0.01), tubular interstitial fibrosis and tubular atrophy (IFTA) scores (*r* = 0.510, *P* = 0.007), and negatively correlated with eGFR (*r* = −0.388, *P* = 0.012). These findings revealed that TLR4 and GSDMD may be involved in the development of DKD.

### Inhibition of TLR4 Protects Tubular Cell Injury by Suppressing GSDMD-Related Pyroptosis in db/db Mice

To explore whether *in vivo* inhibition of the TLR4 influences tubular injury and GSDMD-mediated pyroptosis under diabetic conditions, we examined GSDMD-NT, caspase-1 p20, and IL-18 expression in renal tissues by western blot or immunohistochemistry staining, and the level of IL-1β in kidney cortex by ELISA.

**Figure 2:**
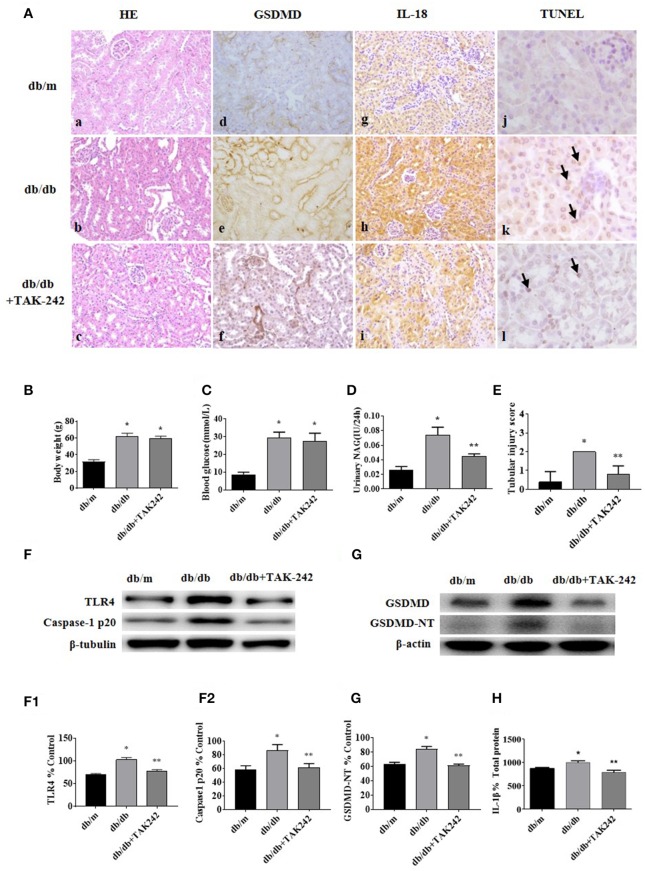
Inhibition of TLR4 protects tubular cell injury by suppressing GSDMD-related pyroptosis in db/db mice. **(A)** Kidney sections stained with HE in db/m (a), db/db (b), and db/db mice treated with TLR4 inhibitor (TAK-242, 3 mg/kg) for 7 days (c); (d–f): IHC staining of GSDMD in renal tissues (magnification ×200); (g–i): representative renal cortical sections of IL-18 IHC staining (magnification ×200); (j–l): representative images of TUNEL staining (arrow show) (magnification ×400). **(B)** Body weight changes in different groups. **(C)** Blood glucose levels. **(D)** Urinary NAG levels. **(E)** Tubular injury scores. **(F)** Western blotting and densitometric analysis of TLR4 (F1) and caspase-1 p20 (F2) expressions in kidney tissues of db/m, db/db, and db/db mice treated with TAK-242. **(G)** WB and densitometric analysis (G1) of GSDMD-NT in kidney tissues in different groups. **(H)** IL-1β level in kidney homogenate was measured by ELISA. Each assay was representative of three independent experiments. Data were expressed as means ±SEM. (*P < 0.05 compared with db/m group, **P < 0.05 compared with db/db group).

As shown in Figure 2Aa–c, increased detachment of renal tubular brush border, tubular atrophy, and interstitial fibrosis in db/db mice was seen when compared with the control group, which were ameliorated by the injection of TLR4 inhibitor TAK-242. IHC staining of GSDMD (Figure 2Ad–f) and IL-18 (Figure 2Ag–i) showed increased expression predominately located in renal tubular cells in db/db mice, which decreased after TAK-242 treatment. Increased cell death was seen in tubular cells of diabetic mouse kidneys by TUNEL staining (Figure 2Aj–l), while the effect was alleviated by TLR4 inhibitor treatment.

There was no significant difference in body weight and blood glucose levels between db/db mice group and db/db+TAK-242 group (Figures 2B,C). As seen in Figure 2D, urinary excretion of β-NAG, the marker of tubular injury, was consistently increased in diabetic mice. Treatment with TAK-242 significantly lowered the elevated urinary β-NAG level. The same changes also happened in tubular injury score (Figure 2E), indicating improvement of tubular function after TLR4 inhibition.

We further detected the protein levels of Caspase-1 p20 and GSDMD-NT in renal cortex tissue. Both of which were increased in db/db mice and obviously decreased after TAK-242 injection (Figures 2F,G). IL-1β level in kidney homogenates (Figure 2H) was upregulated in db/db mice and decreased after TAK-242 treatment. These findings revealed that TLR4 inhibition distinctly suppressed GSDMD-related pyroptosis and alleviated renal injury in db/db mice.

### TLR4/ NF-κB and GSDMD-NT Expressions Were Increased in HK-2 Cells Under HG Ambience

**Figure 3:**
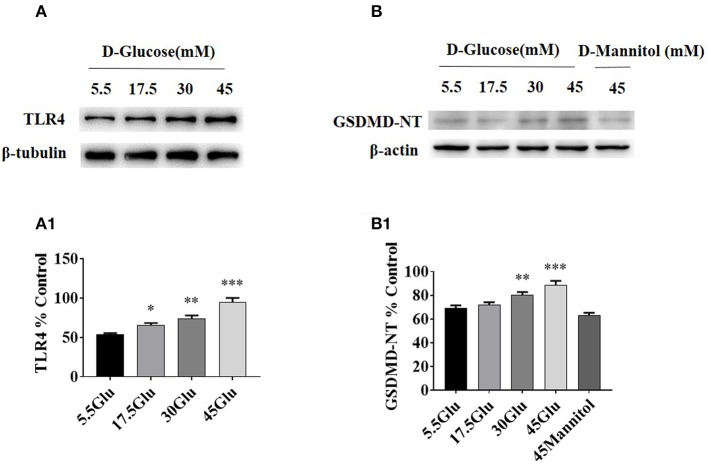
TLR4/ NF-κB and GSDMD-NT expressions were increased in HK-2 cells under HG condition. **(A)** WB and densitometric analysis of TLR4 in HK-2 cells exposed to different concentrations of D-glucose. **(B)** Protein expression of GSDMD-NT exposed to different concentrations of D-glucose. The protein level of TLR4 (A1) and GSDMD-NT(B1) in HK-2 cells exposured to D-glucose (5.5, 17.5, 30, and 45 mM) for 24 h. Each assay was representative of three independent experiments. Data were expressed as means ±SEM. (*P < 0.05 vs. control; **P < 0.05 vs. 17.5Glu group; ***P < 0.05 vs. 30Glu group).

To dissect the effect of high glucose on tubular TLR4 and GSDMD-NT expression *in vitro*, we exposed cultured HK-2 cells to D-glucose (15–45 mM) for 24 h and showed an upregulation of TLR4 (Figure 3A) and GSDMD-NT expression in a dose-dependent manner, whereas exposure to an equivalent dose of mannitol (45 mM) had no effect on GSDMD-NT expression (Figure 3B).

### Inhibition of TLR4/NF-κB Signaling Decreased the Expression of Caspase-1 and GSDMD-NT in HK-2 Cells Under HG Ambience

Due to the similar change of TLR4 and GSDMD-NT in HK-2 cells under different concentration of high glucose, we next examined the effect of TLR4 or NF-κB inhibition on pyroptosis-related protein. The levels of p-p65, p65, Caspase-1, Caspase-1 p20, GSDMD, and GSDMD-NT were determined by western blot, and ELISA was performed to detect the level of IL-1β in supernatant of HK-2 cells.

**Figure 4:**
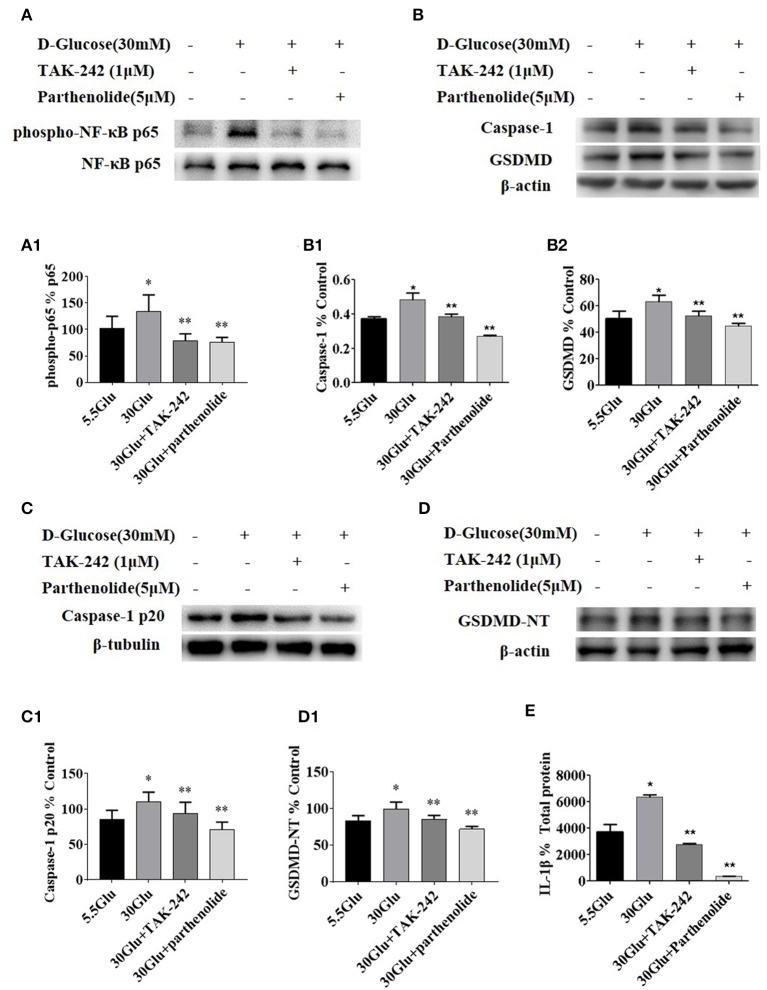
Inhibition of TLR4/NF-κB signaling decreased the expression of caspase-1 and GSDMD in HK-2 cells under HG condition. **(A)** HK-2 cells were treated with TLR4 inhibitor (TAK242, 1 μM) for 2 h prior to HG (30 mM) treatment and with NF-κB blocker (parthenolide, 5 μM) and HG (30 mM) for 2 h, phospho-NF-κB p65 increased in 30 mM HG for 2 h compared to the control, while TAK-242 and parthenolide alleviated HG-induced NF-κB p65 phosphorylation. **(B–D)** HK-2 cells were treated with TLR4 inhibitor (TAK-242, 1 μM) for 2 h prior to HG (30 mM) treatment for 24 h or with NF-κB blocker (parthenolide, 5 μM) and HG (30 mM) for 24 h, and samples were collected for Western blot analysis to detect caspase-1 (B1), GSDMD(B2), caspase-1 p20 (C1) and GSDMD-NT(D1) expressions. **(E)** IL-1β level in supernatant of HK-2 cells measured by ELISA was increased in 30 mM glucose group and reversed both in 30GLU+ TAK242 and 30GLU+ parthenolide groups. Each assay was representative of three independent experiments. Data were expressed as means ±SEM. (*P < 0.05 vs. control group; **P < 0.05 vs. 30Glu group).

The results demonstrated that the expression levels of p-p65, Caspase-1 p20, and GSDMD-NT were increased in HG-stimulated HK-2 cells than that in control group. However, TLR4 or NF-κB inhibition led to a significant decrease of p-p65, Caspase-1 p20, and GSDMD-NT in HG-stimulated HK-2 cells (Figure 4), indicating that NF-κB was the downstream signal molecules of TLR4 under HG ambience and the activation of pyroptosis in HK-2 cells was markedly inhibited by TLR4/NF-κB signaling inhibition.

### Inhibition of TLR4/NF-κB Signaling Alleviated Pyroptosis in HK-2 Cells Under HG Ambience

**Figure 5:**
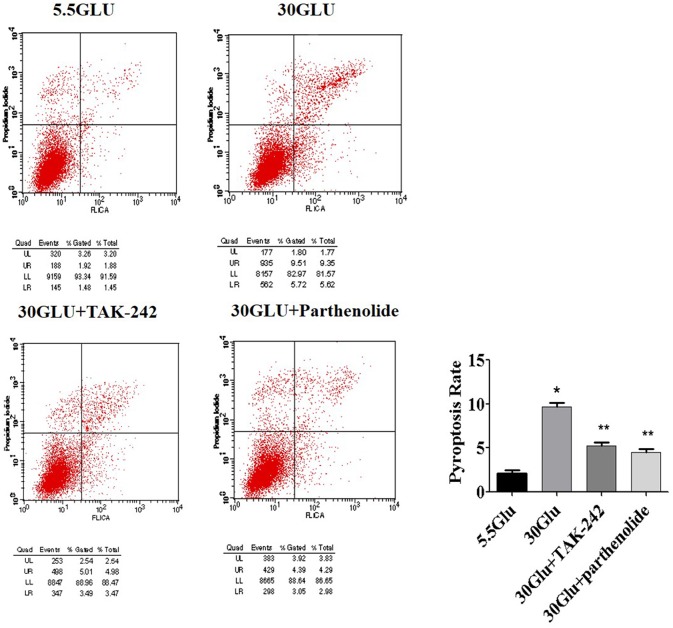
Inhibition of TLR4/NF-κB signaling reversed pyroptosis in HK-2 cells under HG ambience. Right upper quadrant presented pyroptosis of HK-2 cells, flow cyometry analysis show high glucose (9.35%) increased HK-2 cells pyroptosis significantly compared to the control (1.88%), and was reversed both in 30GLU+ TAK242 (4.98%) and 30GLU+ parthenolide groups (4.29%). Each assay was representative of three independent experiments. Data were expressed as means ±SEM. (*P < 0.05 vs. 5.5Glu group; **P < 0.05 vs. 30Glu group).

Pyroptosis is a caspase-1-dependent regulated cell death. To verify the necrotic nature of high glucose induced pyroptosis, caspase-1 activation in HK-2 cells were detected by labeling the cells with PI and Alexa Fluor 488 labeled caspase-1 FLICA. Double positive cells were considered to be pyroptotic cells. As shown in Figure 5, high glucose induced an increase in HK-2 cells pyroptosis compared to the control. However, TLR4 or NF-κB blockers treatment alleviated these changes, suggesting an essential role of TLR4/NF-κB pathway in pyroptosis in HG-stimulated HK-2 cells.

## Discussion

In this study, we identified that TLR4/NF-κB signaling could activate GSDMD-related pyroptosis in high glucose ambience. We have proved that GSDMD, which is the key factor in the process of pyroptosis, had a close correlation with tubular injury. In addition, TLR4/NF-κB signaling might involve in the mechanism of GSDMD-related tubular pyroptosis in high glucose ambience during the progression of DKD.

Researches have been proved that TLR4-mediated pathway play an essential role in the progression of DKD (11, 16). In addition, several evidences have been shown that TLRs activation can trigger inflammasome formation and cell death pyroptosis, which is dependent on caspase-1 (17–20). These findings promote us to detect the expression of TLR4 in DKD patients and analyze the correlation between them in tubular cells. We detected the increased expression of TLR4 in tubular cells was related with increased tubular injury (Figure 1). *In vivo* and *in vitro*, high-glucose induced increased expression of TLR4, cleaved caspase-1, GSDMD and secretion of IL-1β, which could partly reversed by TLR4 and NF-κB inhibitors. In addition, the inhibition of TLR4 could reduce the activation of NF-κB, and inhibitor of NF-κB (parthenolide) could also ameliorate the pyroptosis level of HK-2 cells under the high glucose condition. These results indicated that TLR4/NF-κB signaling modulates pyroptosis in tubular cells in DKD.

Recently, GSDMD has been identified to be one of the key proteins in caspase-1-mediated pyroptosis and controlling IL-1β release in mouse bone marrow macrophages (21, 22). Activated caspase-1 cleaves GSDMD to generate an N-terminal cleavage product (GSDMD-NT) that triggers pyroptosis. But there is no research focusing on whether GSDMD also contributes to the development of pyroptosis in DKD. In this study, we have detected strong expression of GSDMD in the tubulars of DKD patients. Diabetic mice model and HK-2 cells experiments show that the inhibition of TLR4/NF-κB signaling could reverse the increased expression of GSDMD-NT under the high glucose ambience, accompanied with the inhibition of pyroptosis and the release of IL-1β. Thus, TLR4/NF-κB signaling was proved to be involved in GSDMD-related pyroptosis in DKD.

## Conclusion

TLR4/NF-κB signaling could activate GSDMD-related pyroptosis in tubular cells *in vitro* and *in vivo*, hoping to provide a better understanding and more effective therapeutic approach for the prevention and treatment of DKD.

## Data Availability

The raw data supporting the conclusions of this manuscript will be made available by the authors, without undue reservation, to any qualified researcher.

## Ethics Statement

Kidney biopsy sections from patients diagnosed with DN (*n* = 30) and non-diabetic patients diagnosed as minor glomerular lesions (N-DKD, *n* = 13) were obtained from the second Xiangya Hospital of Central South University following the Institutional Review Board of the Second Xiangya Hospital. Written informed consent was obtained from all the participants. Animal experiments were approved by the institutional committees of the Animal Research Committee and Animal Ethics Committee of the Second Xiangya Hospital.

## Author Contributions

YW revised the data, edited the manuscript, and completed submission. XZ conceived and supervised the study. SY reviewed the manuscript and helped interpreted the histology data. SW performed most experiments and drafted the manuscript. XL guided the cell experiments. ZQ and CW helped in animal experiments. JL and HL helped designed the experiments. FL provided guidance to the study. All authors read and approved the manuscript.

### Conflict of Interest Statement

The authors declare that the research was conducted in the absence of any commercial or financial relationships that could be construed as a potential conflict of interest.

## References

[1] Zhu X Xiong X Yuan S Xiao L Fu X Yang Y. Validation of the interstitial fibrosis and tubular atrophy on the new pathological classification in patients with diabetic nephropathy: a single-center study in China. J Diabetes Complications. (2016) 30:537–41. 10.1016/j.jdiacomp.2015.12.002 26796433

[2] Xiao L Zhu X Yang S Liu F Zhou Z Zhan M. Rap1 ameliorates renal tubular injury in diabetic nephropathy. Diabetes. (2014) 63:1366–80. 10.2337/db13-1412 24353183 PMC3964498

[3] Orrenius S Nicotera P Zhivotovsky B. Cell death mechanisms and their implications in toxicology. Toxicol Sci. (2011) 119:3–19. 10.1093/toxsci/kfq268 20829425

[4] Fink SL Cookson BT. Caspase-1-dependent pore formation during pyroptosis leads to osmotic lysis of infected host macrophages. Cell Microbiol. (2006) 8:1812–25. 10.1111/j.1462-5822.2006.00751.x 16824040

[5] Li X Du N Zhang Q Li J Chen X Liu X. MicroRNA-30d regulates cardiomyocyte pyroptosis by directly targeting foxo3a in diabetic cardiomyopathy. Cell Death Dis. (2014) 5:e1479. 10.1038/cddis.2014.430 25341033 PMC4237254

[6] Zhang Z Shao X Jiang N Mou S Gu L Li S. Caspase-11-mediated tubular epithelial pyroptosis underlies contrast-induced acute kidney injury. Cell Death Dis. (2018) 9:983. 10.1038/s41419-018-1023-x 30250284 PMC6155357

[7] Yang JR Yao FH Zhang JG Ji ZY Li KL Zhan J. Ischemia-reperfusion induces renal tubule pyroptosis via the CHOP-caspase-11 pathway. Am J Physiol Renal Physiol. (2014) 306:F75–84. 10.1152/ajprenal.00117.2013 24133119

[8] An H Qian C Cao X. Regulation of Toll-like receptor signaling in the innate immunity. Sci China Life Sci. (2010) 53:34–43. 10.1007/s11427-010-0011-x 20596954

[9] Anders HJ Banas B Schlondorff D. Signaling danger: toll-like receptors and their potential roles in kidney disease. J Am Soc Nephrol. (2004) 15:854–67. 10.1097/01.asn.0000121781.89599.16 15034087

[10] Chen J John R Richardson JA Shelton JM Zhou XJ Wang Y. Toll-like receptor 4 regulates early endothelial activation during ischemic acute kidney injury. Kidney Int. (2011) 79:288–99. 10.1038/ki.2010.381 20927041 PMC3404515

[11] Lin M Yiu WH Wu HJ Chan LY Leung JC Au WS. Toll-like receptor 4 promotes tubular inflammation in diabetic nephropathy. J Am Soc Nephrol. (2012) 23:86–102. 10.1681/ASN.2010111210 22021706 PMC3269929

[12] Wei M Li Z Xiao L Yang Z. Effects of ROS-relative NF-kappaB signaling on high glucose-induced TLR4 and MCP-1 expression in podocyte injury. Mol Immunol. (2015) 68:261–71. 10.1016/j.molimm.2015.09.002 26364141

[13] Yang S Zhang J Wang S Zhao X Shi J. SOCS2 overexpression alleviates diabetic nephropathy in rats by inhibiting the TLR4/NF-kappaB pathway. Oncotarget. (2017) 8:91185–98. 10.18632/oncotarget.20434 29207635 PMC5710915

[14] Napier BA Brubaker SW Sweeney TE Monette P Rothmeier GH Gertsvolf NA. Complement pathway amplifies caspase-11-dependent cell death and endotoxin-induced sepsis severity. J Exp Med. (2016) 213:2365–82. 10.1084/jem.20160027 27697835 PMC5068231

[15] Tervaert TW Mooyaart AL Amann K Cohen AH Cook HT Drachenberg CB. Pathologic classification of diabetic nephropathy. J Am Soc Nephrol. (2010) 21:556–63. 10.1681/ASN.2010010010 20167701

[16] Yuan S Liu X Zhu X Qu Z Gong Z Li J. The role of TLR4 on PGC-1alpha-mediated oxidative stress in tubular cell in diabetic kidney disease. Oxid Med Cell Longev. (2018) 2018:6296802. 10.1155/2018/6296802 29861832 PMC5976914

[17] Nystrom S Antoine DJ Lundback P Lock JG Nita AF Hogstrand K. TLR activation regulates damage-associated molecular pattern isoforms released during pyroptosis. EMBO J. (2013) 32:86–99. 10.1038/emboj.2012.328 23222484 PMC3545309

[18] Schroder K Tschopp J. The inflammasomes. Cell. (2010) 140:821–32. 10.1016/j.cell.2010.01.040 20303873

[19] Kawai T Akira S. Toll-like receptors and their crosstalk with other innate receptors in infection and immunity. Immunity. (2011) 34:637–50. 10.1016/j.immuni.2011.05.006 21616434

[20] Warren SE Mao DP Rodriguez AE Miao EA Aderem A. Multiple Nod-like receptors activate caspase 1 during *Listeria monocytogenes* infection. J Immunol. (2008) 180:7558–64. 10.4049/jimmunol.180.11.7558 18490757 PMC2991040

[21] Shi J Zhao Y Wang K Shi X Wang Y Huang H. Cleavage of GSDMD by inflammatory caspases determines pyroptotic cell death. Nature. (2015) 526:660–5. 10.1038/nature15514 26375003

[22] He WT Wan H Hu L Chen P Wang X Huang Z. Gasdermin D is an executor of pyroptosis and required for interleukin-1beta secretion. Cell Res. (2015) 25:1285–98. 10.1038/cr.2015.139 26611636 PMC4670995

